# Cardiovascular Dysfunction Due to Sympathetic Hypoactivity After Complete Cervical Spinal Cord Injury: A Case Report and Literature Review

**DOI:** 10.1097/MD.0000000000000686

**Published:** 2015-03-27

**Authors:** Young-Min Oh, Jong-Pil Eun

**Affiliations:** From the Department of Neurosurgery (Y-MO, J-PE), Research Institute of Clinical Medicine, Chonbuk National University, and Biomedical Research Institute, Chonbuk National University Hospital, Jeonju, Korea.

## Abstract

Spinal cord injury (SCI) is one of the most devastating of all traumatic events; it may cause permanent dysfunction in several organ systems and lead to motor and sensory impairment. Cardiovascular dysfunction has been recognized to be the leading cause of morbidity and mortality in the acute and chronic stages following SCI. Although cardiovascular dysfunction causes the deaths of many SCI patients, most clinicians are unfamiliar with the phenomenon. The purpose of reporting our case is to remind clinicians to consider the possibility of cardiovascular dysfunction in patients with complete SCI.

The patient signed informed consent for publication of this case report and any accompanying image. The ethical approval of this study was waived by the ethics committee of the Chonbuk National University Hospital, Jeonju, Korea, because this study was a case report and the number of patients was <3.

A 63-year-old man was transferred to our emergency room after a fall. He complained of weakness and numbness of the lower extremity. Radiologic evaluation revealed C7/T1 unilateral facet dislocation with spinal cord contusion. On neurologic examination, the patient exhibited a paraplegic state below the T4 dermatome because of complete SCI. His vital signs were stable, but respiration was shallow. We performed intraoperative manual reduction and anterior interbody fusion. On the second postoperative day, the patient experienced sudden cardiac arrest after he was shifted from a supine to a semilateral position. Upon position change, heart rate was decreased <40 beats/min and blood pressure could not be checked. We immediately started cardiac massage and administered atropine 0.5 mg and epinephrine 1 mg, and the patient was successfully resuscitated. Cardiac arrest recurred when we performed endotracheal suction or changed patient's position. Echocardiographic and Holter monitoring findings demonstrated normal heart function and sinus bradycardia, and there was no evidence of pulmonary thromboembolism. We concluded that cardiac arrest was induced by sympathetic hypoactivity following complete SCI.

Two months later, this phenomenon had resolved, and 4 months after presentation, he was discharged reliant on a home ventilator.

Through this report, we emphasize that a thorough understanding of cardiovascular dysfunction following SCI is important for establishing a diagnosis and optimizing clinical outcomes.

## INTRODUCTION

Spinal cord injury (SCI) is one of the most devastating of all traumatic events; it may cause permanent dysfunction in several organ systems and lead to motor and sensory impairment.^1–3^ Renal and respiratory complications have been to be the most frequent adverse events after SCI and the most common causes of death^4^; however, more recently, cardiovascular dysfunction has been recognized to be the leading cause of morbidity and mortality in the acute and chronic stages following SCI.^5,6^ An acute SCI above the sixth thoracic (T6) vertebra disrupts the descending pathways to the sympathetic neurons located in the intermediolateral cell column of the spinal cord of the first thoracic (T1) vertebra through the second lumbar (L2) vertebrae.^7^ Therefore, patients with cervical and high thoracic SCI exhibit impairment in control of the autonomic nervous system (ANS), which causes bradycardia, arterial hypotension, and autonomic dysreflexia. Additional vascular complications, such as deep vein thrombosis and long-term risk for coronary heart disease and systemic atherosclerosis, may occur. Although cardiovascular dysfunction causes the deaths of many SCI patients, most clinicians are unfamiliar with the phenomenon.

Recently, we treated a patient who experienced cardiovascular dysfunction after SCI. Herein, we report the case of this patient and review literature on cardiovascular dysfunction after SCI.

## CASE

**FIGURE 1:**
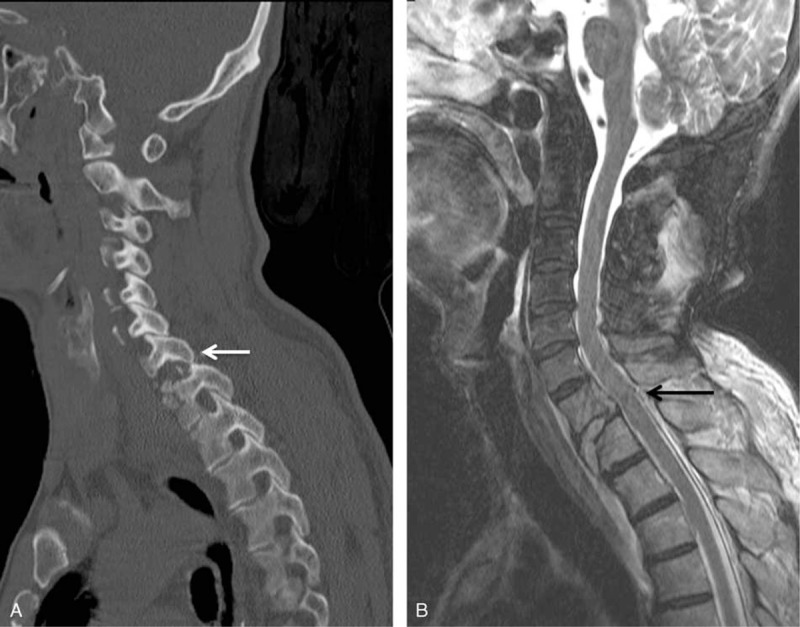
(A) Three-dimensional computed tomography image demonstrating C7/T1 unilateral facet dislocation. (B) Magnetic resonance image demonstrating spinal cord contusion and hemorrhage. There were ruptured disc materials on C7/T1 and rupture of the anterior longitudinal ligament.

A 63-year-old man was transferred to our emergency room after a fall. He complained of weakness and numbness of the lower extremity. Radiologic evaluation revealed C7/T1 unilateral facet dislocation (Figure 1A) and magnetic resonance image demonstrated spinal cord contusion (Figure 1B). On neurologic examination, the patient exhibited a paraplegic state below the T4 dermatome because of complete SCI. His vital signs were stable, but respiration was shallow. We performed intraoperative manual reduction and anterior interbody fusion.

On the second postoperative day, the patient experienced sudden cardiac arrest after he was shifted from a supine to a semilateral position. Upon position change, heart rate was decreased <40 beats/min and blood pressure could not be checked. We immediately started cardiac massage and administered atropine 0.5 mg and epinephrine 1 mg, and the patient was successfully resuscitated. Cardiac arrest recurred when we performed endotracheal suction or changed patient's position. We consulted the cardiology department to investigate these cardiac arrests. Echocardiographic and Holter monitoring findings demonstrated normal heart function and sinus bradycardia, and there was no evidence of pulmonary thromboembolism. We concluded that cardiac arrest was induced by sympathetic hypoactivity following complete SCI.

On the 10th postoperative day, the frequency of these arrests after endotracheal suction or position change decreased, and we prepared atropine before these procedures to enable rapid response. The patient's heart rate was recovered soon after injection of 0.5 mg atropine. Two months later, this phenomenon had resolved, and 4 months after presentation, he was discharged reliant on a home ventilator.

## DISCUSSION

### Epidemiology

All patients with motor complete cervical SCI (American Spinal Injury Association [ASIA]) A and B) develop bradycardia, 68% develop arterial hypotension, 35% require vasopressor treatment, and cardiac arrest occurs in 16%.^8,9^ In patients with motor incomplete cervical SCI (ASIA C and D), 35% to 71% develop bradycardia, but few experience hypotension or require vasopressor treatment, and cardiac arrest is very rare.^8^

### Pathophysiology

The ANS plays an important role in the cardiovascular system. Blood pressure and heart rate are controlled by impulses from the ANS; parasympathetic impulses decrease the heart rate, whereas sympathetic impulses increase heart rate, myocardial contractility, peripheral vascular resistance, and arterial blood pressure by inducing vasoconstriction.^9^

The descending pathways of the sympathetic neurons are located in the intermediolateral cell column of the spinal cord of the first thoracic (T1) vertebra through the second lumbar (L2) vertebrae.^7^ Parasympathetic neurons from the dorsal motor neurons of the vagus and the nucleus ambiguous in the medulla oblongata reach the heart via the recurrent laryngeal nerve and the vagus nerve.^10^ Therefore, the degree of sympathetic cardiovascular dysfunction is directly related to the location and severity of SCI.^11^

In our case, the patient exhibited complete SCI due to unilateral facet dislocation at the C7/T1 level. Secondary to SCI, the descending pathways of the patient's sympathetic nervous system were interrupted. Disruption of these spinal tracts and the intact vagus nerve resulted in sympathetic hypoactivity and unopposed parasympathetic outflow.

### Treatment and Prevention

In most of these patients, bradycardia peaked at day 4 postinjury and then gradually resolved over the following 2 to 3 weeks.^12^ Because efferent cardiac parasympathetic nerve pathways remain intact in the presence of reduced sympathetic activity, it is important to avoid increase in the vagal reflexes during this period, such as that induced by position change and endotracheal suction. Hypoxia, hypoventilation, and the presence of tubes in the nose or mouth may cause bradycardia mediated by increased vagal reflexes.^13^ Atropine should be kept readily available, as necessary. Previously, Hagen et al^7^ suggested that the first-line therapy for bradycardia is dopamine. Atropine and a temporary pacemaker are used as the second-line therapy if the patient does not respond to dopamine. Implantation of a permanent pacemaker may be necessary in prolonged or excessive symptomatic bradycardia. In addition, adequate oxygenation is vital for the prevention of bradycardia and cardiac arrest. Atropine should be administered before any tracheal procedures.^14^

## CONCLUSION

Cardiovascular dysfunctions commonly occur following severe SCI. Loss of supraspinal control of the sympathetic nervous system is the major cause of cardiac dysfunction in patients with severe SCI. These physiologic changes are much more common in patients with motor complete SCI at or rostral to T6. A thorough understanding of cardiovascular dysfunction following SCI is important for establishing a diagnosis and optimizing clinical outcomes.
